# Differential determinants of virulence in two *Mycobacterium tuberculosis* Colombian clinical isolates of the LAM09 family

**DOI:** 10.1080/21505594.2019.1642045

**Published:** 2019-07-22

**Authors:** Andres Baena, Felipe Cabarcas, Karen L.F. Alvarez-Eraso, Juan Pablo Isaza, Juan F. Alzate, Luis F. Barrera

**Affiliations:** a Grupo de Inmunología Celular e Inmunogenética (GICIG), Facultad de Medicina, Universidad de Antioquia, Medellín, Colombia; b Departamento de Microbiología y Parasitología, Facultad de Medicina, Universidad de Antioquia, Medellín, Colombia; c Centro Nacional de Secuenciación Genómica (CNSG), Facultad de Medicina, Universidad de Antioquia, Medellín, Colombia; d Grupo SISTEMIC, Ingeniería Electrónica, Facultad de Ingeniería, Universidad de Antioquia, Medellín, Colombia; e Grupo de Parasitología, Facultad de Medicina, Universidad de Antioquia, Medellín, Colombia; f Instituto de Investigaciones Médicas, Facultad de Medicina, Universidad de Antioquia, Medellín, Colombia

**Keywords:** Mycobacterium tuberculosis, virulence, RNA-seq, transcriptome, clinical isolates, Colombia

## Abstract

The heterogeneity of the clinical outcome of *Mycobacterium tuberculosis* (Mtb) infection may be due in part to different strategies used by circulating strains to cause disease. This heterogeneity is one of the main limitations to eradicate tuberculosis disease. In this study, we have compared the transcriptional response of two closely related Colombian clinical isolates (UT127 and UT205) of the LAM family under two axenic media conditions. These clinical isolates are phenotypically different at the level of cell death, cytokine production, growth kinetics upon in vitro infection of human tissue macrophages, and membrane vesicle secretion upon culture in synthetic medium. Using RNA-seq, we have identified different pathways that account for two different strategies to cope with the stressful condition of a carbon-poor media such as Sauton’s. We showed that the clinical isolate UT205 focus mainly in the activation of virulence systems such as the ESX-1, synthesis of diacyl-trehalose, polyacyl-trehalose, and sulfolipids, while UT127 concentrates its efforts mainly in the survival mode by the activation of the DNA replication, cell division, and lipid biosynthesis. This is an example of two Mtb isolates that belong to the same family and lineage, and even though they have a very similar genome, its transcriptional regulation showed important differences. This results in summary highlight the necessity to reach a better understanding of the heterogeneity in the behavior of these circulating Mtb strains which may help us to design better treatments and vaccines and to identify new targets for drugs.

## Introduction

Tuberculosis (TB) is a contagious infectious disease that in 2017 was responsible for more deaths (1.6 million people) than HIV and malaria [1]. In the same year, there were 10 million new TB cases worldwide, of which 1 million were children. It is estimated, that one-third of the world’s population is infected with *Mycobacterium tuberculosis* (Mtb), the etiological agent of TB. Although the majority of cases (two thirds) are concentrated in eight countries (India, Indonesia, China, Nigeria, Pakistan, Philippines, Bangladesh, and South Africa) [1], least populated countries as Colombia are showing an increasing number of cases, specifically in the vulnerable, marginal populations [2]. In 2017, Colombia notified 16,000 new cases of TB with an incidence of 33 cases per 100,000 inhabitants [1,2]. The county of Antioquia has one of the highest number of incident cases and its capital city Medellin showed an increase from 50 cases to 70 cases per 100,000 individuals in 2016, but in some vulnerable populations such as homeless people, the incidence reaches 7.9% [3]. What is worrisome is that the incidence of TB in prisons and some native Indian communities in Colombia are in the order of 500–600 cases per 100,000 inhabitants, which is comparable to some of the major TB incident countries mentioned above [4].

A main feature of TB, is its high heterogeneity in the disease course and the response to therapy, primarily associated with variability in host determinants but also related to strain variation [5]. This heterogeneity that exists among Mtb strains has an impact on immunogenicity and virulence. The next-generation sequencing technologies is a tool that allows us to have a better understanding of this strain heterogeneity.

Ratified by many studies, the human Mtb associated strains have been clustered in seven main phylogenetic lineages [6,7], and increasing evidence suggests that these lineages have significant phenotypic and virulent consequences [8]. Different clinical isolates vary considerably in their genetic repertoire and manifest in a variety of distinctively clinical profiles in the context of different human populations [9]. The majority of Mtb strains could be classified in to four main lineages apart from the clades of *M. africanum*. Lineage 4 (L4) contains the families Haarlem, Latin-American-Mediterranean (LAM), T, and X. The L4 is one of the most widely distributed and one of the main contributors to the burden of TB patients in the world [8]. The LAM family is the most frequently isolated from TB patients of Colombia [10]. Inside the LAM family, there are different subgroups with variable virulence, which is manifested in different clinical isolates [11]. A previous report from our laboratory, based on phenotypic differences of human monocytes and macrophages in response to infection with two Colombian LAM09 (UT127 and UT205) clinical isolates, suggested differences in virulence between these closely related strains [12]. These clinical isolates were obtained from a study of a cohort of 2060 household contacts of about 438 smear positive pulmonary TB cases that were gathered in Medellın from 2005 to 2009 [13]. One of the main observations that we obtained from this cohort, was that even though the transmission of Mtb occurred to many household contacts, just a few of them developed the active pulmonary disease. We chose the clinical isolate UT127 as a strain type that did not develop an active disease case in its household contacts and on the other hand, we choose the clinical isolate UT205 as a strain that did develop an active disease case in its household contacts. Also, the clinical isolate UT205 was associated with an increased cell death that involved cell membrane damage in IFNγ -treated human splenic macrophages, in contrast to the infection of macrophages with UT127 that resulted in a more apoptotic cell death type [12]. In this paper, we found additional phenotypic differences between these two clinical isolates related to growth kinetics and membrane vesicle secretion (MVs). Notably, we concluded that although both clinical isolates belonged to the same Mtb LAM09 family, their transcription programs are substantially different suggesting that these strains may have evolved alternative genetic programs in order to adapt to host environmental changes.

## Materials and methods

### Bacterial strain and culture conditions

For the axenic cultures, Mtb clinical isolates UT127, UT205 and H37Rv were grown at 37°C in a shaker at 120 rpm in 10 ml of 7H9 (Difco, Sparks, MD) supplemented with 10% of oleic acid-albumin-dextrose-catalase (OADC) (Becton Dickinson Microbiology Systems, New Jersey, USA), 0.5% Glycerol (Sigma, Saint Louis, MO, USA) and 0.05% of Tyloxapol (Sigma, Saint Louis, MO, USA), in an 30 ml square bottle to an optical density of 0.5 at OD_600_ nm. The bacteria were pelleted by centrifugation and 600 million bacteria were re-suspended in fresh 7H9 or Sauton’s media (KH_2_PO_4_, Na_2_HPO_4_, asparagine, ferric ammonium citrate, MgSO_4_, CaCl_2_, ZnSO_4_, Tyloxapol, and glycerol) [14], containing all supplements in the case of 7H9, and grown with agitation as before for 6 hrs. UT127 and UT205 were genotyped by spoligotyping, and both strains are SIT42 (octal code 777,777,607,760,771) that correspond to LAM9. This was further confirmed by MIRUs (UT127-124,326,153,220 and UT205- 224,226,153,321). Furthermore, UT127 is 4.3.3, and UT205 is 4.3.4.2 based on the barcode classification [15].

### Growth curve of axenic cultures

The two Mtb clinical isolates UT127 and UT205 and the reference strain H37Rv were grown aerobically at 37°C in 7H9 (BD Middlebrook, Difco, Sparks, MD) supplemented with 10% (v/v) OADC (Becton Dickinson Microbiology Systems, New Jersey, USA), 0.5% (v/v) glycerol and 0.05% (v/v) Tyloxapol and Sauton’s medium as mentioned above. Growth was monitored daily by OD absorbance at 600 nm for 10 days. Results are representative of two independent experiments.

### Membrane vesicle (MVs) isolation

To isolate the MVs, the bacteria for each clinical isolate were grown in a Sauton´s minimal medium (Identical to Sauton’s medium but without OADC and Tyloxapol) for 15 days. Vesicles were isolated, as previously described [16]. Briefly, cells were pelleted (3,000 rpm for 10 min at 4°C) from 500-ml cultures, and the supernatants were ﬁltered through a 0.45-µm ﬁlter (Merck-Millipore, Billerica, MA, USA). The supernatant volumes were then concentrated using an Amicon (Merck-Millipore, Billerica, MA, USA) ultraﬁltration system with a 100-kDa ﬁlter. The concentrate was then centrifuged at 55,000 rpm for 2 h at 4°C to sediment the vesicular fraction into a pellet. The supernatant was discarded, and the pellet was suspended in PBS. The quantification was done by calculating the total protein content in the MVs using the BCA kit (Bio-Rad, Hercules, CA, USA).

### Genome of UT127

Mtb UT127 was isolated from the sputum of a 35‐year‐old man with pulmonary tuberculosis. A single colony from Dubos solid medium was transferred to 7H9 liquid medium supplemented with OADC and Tween‐80, cultured to an OD600 nm of ~0.5, harvested by centrifugation and resuspended in TE pH 8.0. For genomic DNA extraction, mycobacteria were freeze‐thawed in ethanol‐dry ice, heated at 80°C, digested with lysozyme and incubated 1 h with 10% SDS at 60°C, and again submitted to five cycles of freeze‐thawing. Genomic DNA was phenol/chloroform/isoamyl alcohol (25:24:1, v/v) extracted, precipitated with isopropanol, washed with 75% ethanol and finally resuspended in TE pH 8.0. Whole genome shotgun sequencing was carried out using the ROCHE 454‐GS‐FLX TITANIUM technology at the National Center for Genomic Sequencing‐CNSG (Medellin‐Colombia), following standard protocols. DNA library construction was performed with the ROCHE’s GS Rapid Library Rgt/Adaptors Kit following all the standard procedures. DNA was fragmented by nebulization, purified, and adapter-ligated as indicated by the manufacturer instructions. Quality of the library was assed using a Bioanalyzer 2100 instrument (AGILENT). The genome assembly process was performed using the Newbler v2.9 software [17], with default settings. Contig reordering and joining were carried out with the ABACAS algorithm [18], based on the H37Rv reference genome (EMBL accession number AL123456). Genome annotation was performed with the RATT tool from the SANGER institute [19], based on the latest H37Rv reference genome (EMBL accession number AL123456). Artemis tool was used to inspect the transferred gene models and to curate those require modifications manually [20]. For the SNV (single nucleotide variations) and large sequence polymorphisms (LSP, ≥50 bases in length) detection, the program NUCmer, from the mummer package v4, was used. The reference was always the above-mentioned H37Rv reference genome. The generated SNPs file was used for the SNV detection. Then the program SIFT [21] was used to predict the genes affected and its consequence on the respective protein. The delta file obtained with NUCmer was loaded into the ASSEMBLYTICS server [22], in order to obtain the LSP features for each strain (https://www.ncbi.nlm.nih.gov/bioproject as a genomic DNAseq bio-project with the code SRR8696725). The genome sequence of UT205 was published previously to this paper [23].

### RNA-seq procedure of axenic cultures

Total RNA, from mycobacterial cultures of UT127, UT205, and H37Rv, was prepared as follow. At the end of the 6 hrs of incubation, the bacterial cultures were incubated on ice for 5 minutes and then pelleted by centrifugation at 3,000 rpm in a refrigerated tabletop centrifuge (4°C) for 10 minutes. The media culture supernatants were discarded, and the pellets were re-suspended in 1 ml of RLT buffer (RNeasy Plus mini kit, QIAGEN, Hilden, Germany). Then the pellets were frozen in liquid nitrogen for 15 seconds and homogenized two times with a tissue tearor homogenizer (Biospec, Bartlesville, OK, USA; model 985–370 at 5,000 rpm) for 20 seconds. The homogenization was done two times with incubation of 1 minute on ice between cycles. Then, bacterial cells were transferred to a 1.5-ml screw cap tube where they were disrupted by bead beating (Bead beater Instrument; six cycles of 30 s at maximum speed with cooling on ice between cycles) with high impact zirconium-silica beads (BenchmarkScientific, Bartlesville, OK, USA). After that, the samples were centrifuged at 4°C/10,000 rpm for 10 min. Finally, the total RNA was extracted from the aqueous phase using the Qiagen RNeasy plus mini kit, including the DNA retention column according to the manufacturer’s instructions. For each condition, three independent experiments were performed, and the extracted RNA was pooled with its related replicates. The RNA RIN values for all the samples were between 8.1 and 8.6.

For the RNA-seq experiment, rRNA was depleted with the Ribo-Zero rRNA Removal Kit (Bacteria) (Illumina CAT# MRZB12424) and used for the preparation of a stranded Illumina Truseq RNA SBS Kit v3 library. The RNA-seq libraries were pooled and ran in a HiSeq2000 lane with PE reads of 100 bases at Macrogen (Seoul, Korea). Raw reads were trimmed to Q35 and retaining the reads that showed at least 70 bases in length. Singleton reads (also called orphan reads) were excluded from the “clean read” dataset. Ribosomal RNA contamination was measured by counting the reads that mapped to the ribosomal cluster of the H37Rv reference genome; in all cases, it was below 8%.

### RNA-seq data analysis

The “clean” read dataset was mapped to the reference genome of the strain H37Rv of *M. tuberculosis* (AL123456_update130713) using the program BOWTIE2 with default parameters. The SAM file was converted to BAM, sorted and indexed using SAMTOOLS. Reads counts assign to each gene model of the reference was performed with HTSEQ script with the *stranded* option. For the differential expression analysis, the R package EDGER was used, following the protocols described by the authors of the software for RNA-seq experiments. Due to the lack of library replicates, the coefficient of biological variation was manually set to 0.3. EDGER analysis results were printed as graphs and tables and saved. The transcriptomic data from the RNA-seq of the laboratory strain H37Rv was deposited in https://www.ncbi.nlm.nih.gov/bioproject under the codes: SRR8695352 and SRR8695353 for UT205, SRR8697251, and SRR8697252 for H37Rv and finally SRR8696726 and SRR8696727 for UT127. The first code number for each Mtb strain refers to 7H9 media and the second for Sauton’s media.

### Quantitative real-time PCR (qRT-PCR)

Independent RNA was isolated from UT205-7H9 and UT205-Sauton´s medium samples as previously described in this section. RNA integrity was assayed by agarose gel electrophoresis, and the concentration was determined by Nanodrop 2000 spectrophotometer (Thermo Scientific, Waltham, MA). The RNA was treated with DNase I (Thermo Scientific, Waltham, MA) to eliminate any potential DNA contamination and then purified by the RNAeasy kit (Qiagen). The cDNA was prepared using the Superscript IV (Thermo Fisher Scientific, Waltham, MA) using random primers according to the manufacturer’s instructions. The qPCR was performed using the Rotor-gene Q machine (QIAgene, Hilden, Germany) and the SYBR Green ER qPCR-master-mix (Thermo Fisher Scientific, Waltham, MA). The experiments were done in triplicates. We used the following primers for the RNAseq validation: *rv1130*-F 5ʹ-TGAAGTGATCGTGGACGAACTG-3ʹ, *rv1130*-R 5ʹ-CTGTTCAACGGGTTCCACTACA-3ʹ, *rv1131*-F 5ʹ-ATTCGTTGACCTACCGGGGATA-3´, *rv1131*-R 5ʹ-CAGCATCGAGCGGTCCAC-3ʹ, frdA-F 5ʹ-ATGGGCTATGACGAGTGGTT-3ʹ, *frdA*-R 5ʹ-GTCTTGATGTTCGCGTTGGT-3ʹ, *rv2485c*-F 5ʹ-TCACCAACGCCGAGAATATG-3ʹ, *rv2485c*-R 5ʹ-CGCTGTGGACGTACGAAAT-3ʹ, *rv3743c*-F 5´-TACAGCGAAGGACCCTAGAT-3ʹ, *rv3743c*-R 5ʹ-GGTTGCGAAGATGACAATGAG-3ʹ, *rpfA*-F 5ʹ-GGTGTGCGGCCGCGGGTTATCG-3ʹ, *rpfA*-R 5ʹ-CCAGCGGTGCGGGCAGGTCGTTAG-3ʹ, *sigA*-F 5ʹ-CATGGTCGAGGTGATCAACAA-3ʹ, *sigA*-R 5ʹ-GGGTGATGTCCATCTCTTTGG-3ʹ and all of these primers were designed with the IDT software (https://www.idtdna.com). We used sigA as a house-keeping gene for the establishment of the fold change of the qRT-PCR.

## Results

### UT205 strain grows better in Sauton’s medium and secretes more MVs than UT127

**Figure 1.**
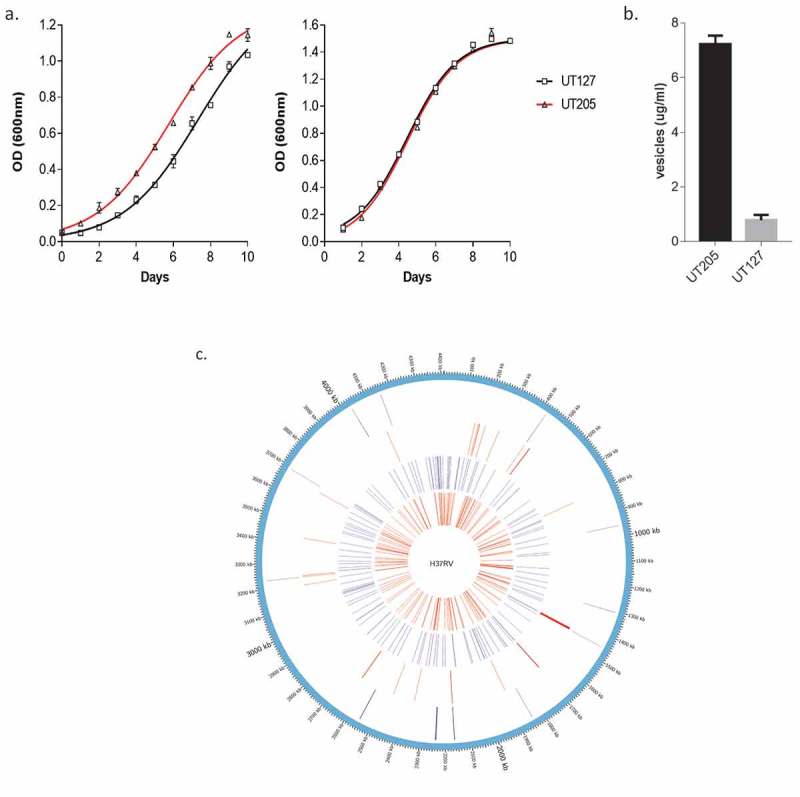
Phenotypic and genotypic changes observed between UT127 and UT205 in axenic cultures. (a). Growth curves of UT127 and UT205 in Sauton’s and Middlebrook 7H9 broth media over 10 days, measured at an optical density (OD) of 600nm. (b). Membrane vesicle (MVs) secretion for UT205 and UT127 measured as a correlation to the total protein content. Both graphs are representative experiments of a series of three independent experiments performed. Each data point represents the average of three measurements. Standard deviation bars are showed for each graph. (c). Graphical representation of the H37Rv chromosome with variations observed in each of the Colombian studied strains, UT205, and UT127, was performed with CIRCOS package [87]. From outside to inside, LSPs UT205, LSPs UT127, SNPs and short variants UT205 and SNPs and short variants UT127.

Previous reports suggested that variations in replication rates could reflect differences in virulence among different Mtb strains [24–29]. Recently, our laboratory provided evidence suggesting that UT127 and UT205 may have differences in virulence [12]. Consequently, we decided to find out if the Colombian clinical isolates UT127 and UT205 could have significant growth differences. To determine the growth kinetics of these two clinical isolates, we set up three independent cultures in the two synthetic media (7H9 and Sauton’s media), for which optical densities were measured at 600 nm every 24 hours during 10 days (Figure 1(a)). The resulted curves in 7H9 rich medium showed that both strains grew strikingly similar in this condition, which suggests the same capabilities of growth for both strains in a rich carbon source environment. In contrast, UT205 was able to grow significantly better than UT127 in Sauton’s media, suggesting better adaptability to a poor-carbon media.

Previously, it has been established that Mtb can secrete membrane vesicles (MVs) that could be used to promote different immune evasion mechanism stimulating bacterial dissemination and pathogenesis [16,30]. Then, we next sought to investigate the total amount of MVs produced by the clinical isolates UT127 and UT205. MVs were isolated from the supernatants of three independent cultures following the procedure described in the methods section. Notably, we found that the clinical isolate UT205 was able to produce more MVs than UT127 (Figure 1(b)). Taking together these results and previously published data, we could conclude that the clinical isolates UT127 and UT205 are different phenotypically, and these differences may be controlled by different regulatory genetic programs orchestrated inside each Mtb isolate.

### Genomic differences among UT127 and UT205

The genome of the *M. tuberculosis* strain UT127 was sequenced similarly as UT205 using the 454 FLX titanium platform. In total, 219,891 shotgun reads were generated and assembled into 102 contigs (larger than 500 bases). Most of the reads were included within the assembly, 99.33%, and the largest contig spanned 222,760 bases. The N50 value reached 101,151 bases, and the total sum of assembled bases was 4,308,887. The average sequencing depth of the contigs was 40X. To analyze the genome, any variants which were identified in insertion sequence (IS) elements or the PE, PE_PGRS, and PPE gene families were excluded, as these regions are recognized to be prone to false-positive single nucleotide polymorphism (SNP) calls.

Comparing to the H37Rv reference genome, we found 159 unique non-synonymous SNPs and short INDELs for UT127 while just 138 were found for UT205 (Figure 1(c), supplementary table 3 and 4). In both strains, we found unique large deletions (>50 bases) up to 3 kbp in size, compared among them and H37Rv reference. UT127 showed the largest number of deletions with a total of 22 that ranged between 51 and 3108 bp. In the case of UT205, as previously reported, 12 were observed and ranged between 53 and 3650. Twenty-one genes were affected by these deletions in UT127, while 11 were altered in UT205. We found one of the LSP of UT127 located within the CDS of an essential gene, *accE5*, which spans 63 bases (in-frame deletion) that will render the protein 21 amino-acids shorter. Notably from these results is a short deletion (13 nucleotides) in the *kdpD* gene (position 1353 to 1365 of the gene) that creates a frame shift that eliminates the N-terminal of the protein. A deletion in *kdpD* that causes a frameshift was previously described in Beijing strains although of a different length and position [31,32]. There are four Mtb strains deposited in NCBI that belongs to Perú (South America) that have the same deletion of 13-bp in *kdpD* (MDRMA701, SLM036, CSV4519, and M0018684-2) [33]. Deletion in the *kdpD* gene could conduct the development of nonfunctional proteins KdpD and KdpE. It was shown that an Mtb strain deficient in KdpD and KdpE function showed increased virulence in a mouse model of infection [34]. The two-component system (TCS) KdpD/KdpE, has a regulatory role in potassium (K+) transport, and more recently, it has been identified as an adaptive regulator involved in virulence [35]. Moreover, KdpD/KdpE has been implicated in promoting survival and virulence of Mtb through various mechanisms distinct from K+ regulation, but it is not yet clear precisely what the mechanism is or where it is implicated. This feature of kdpD might be involved in the increased virulence associated with the clinical isolate UT205.

### Comparative transcriptome profile of UT205 and UT127 strains

In order to test whether different genetic regulatory programs may govern the observed phenotypic changes between UT127 and UT205, a transcriptomic analysis by RNA-seq of the two clinical isolates growing in 7H9 or Sauton’s media was performed.

We obtained a total of 21ʹ387,091 raw read pairs for UT127 in Sauton’s medium while 34ʹ767,672 reads pairs were generated for the 7H9 culture condition. After the cleaning and trimming processes, the remaining reads pairs were 18ʹ406,822 and 34ʹ636,671 for UT127 in Sauton’s and 7H9 cultures, respectively. In the case of UT205 strain, for the Sauton´s culture condition, 16ʹ889,953 read pairs were obtained, from which 14ʹ581,910 of these passed the quality filters. In the 7H9 culture condition, 21ʹ225,720 quality passed read pairs were obtained from 24ʹ559,469 initial raw read pairs. QC passed read pairs from the different culture condition for both strains were mapped to the H37Rv reference genome with a success rate that ranged between 89% and 98%. As an additional quality parameter, the percent of reads mapped to the ribosomal cassette (rDNA) was established. Approximately 7% of the reads from the different conditions mapped to rDNA, excluding UT127 in Sauton’s culture with an alignment rate of 1.3% (Supplementary Table 1). The number of reads mapped to coding-protein genes ranged from 1 to 274,920. The sum of genes without mapped reads were 19, 17, 70, and 70 for the strain/culture media UT127/7H9, UT127/Sauton’s, UT205/7H9, and UT205/Sauton’s, respectively. Moreover, principal component analysis (PCA) shows that the strain and each culture media condition is distinctly clustered by PC1 (51.12%) and PC2 (30.84) respectively (Supplementary Figure 1).

### Differential gene expression between UT127 and UT205

All the transcriptomes were obtained by culturing the different strains of Mtb for 6 h in 7H9 or Sauton’s liquid medium (see methods for more details). We used the laboratory strain H37Rv as an experimental control (see methods for more details) (Supplementary Figure 3). to compare the transcriptome of UT127 or UT205 in their respective culture medium before making the direct comparison between the clinical isolates. In one hand, we obtained 312 upregulated and 112 downregulated genes for UT127 in 7H9 medium (log_2_FC >1.5 and p-value <0.05) and for UT205, we obtained 108 upregulated and 133 downregulated genes in the same medium (log_2_FC >1.5 and p-value <0.05). On the other hand, we got 292 upregulated and 218 downregulated genes for UT127 as compared to 254 upregulated and 213 downregulated genes for UT205 in Sauton’s medium (log_2_FC >1.5 and p-value <0.05) (Supplementary Table 2).

**Figure 2.**
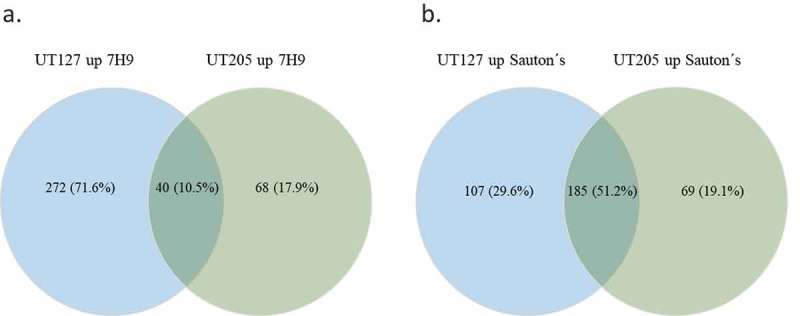
Venn diagram representation of the up-regulated genes among UT127 and UT205. (a). Shows the number and percentage of DE genes for UT127 and UT205 in Middlebrook 7H9 medium and (b). Shows the number and percentage of DE genes for UT127 and UT205 in Sauton’s medium. The UT127 condition appears in blue and UT205 in green. The graphs were obtained using Venny 2.1 software.

When we compared the transcriptome of UT127 to the transcriptome of UT205 in 7H9 medium, we found 272 (71.6%) differentially upregulated genes with a p-value <0.05 that were specific for UT127. One of the most robust observed changes is that UT127 reduced the number of upregulated genes from 272 (71.6%) in 7H9 medium to 107 (29.6%) when it was grown in Sauton’s medium (Figure 2). Remarkably, UT205 did not have any significant changes in the number of upregulated genes in 7H9 vs. Sauton’s media (68 and 69 genes, respectively). This significant reduction in the number of upregulated genes for UT127 but not for UT205 under the Sauton’s medium might represent the effect of a more stressful condition for UT127 than for UT205.

### Main transcriptomic changes between UT127 and UT205

**Figure 3.**
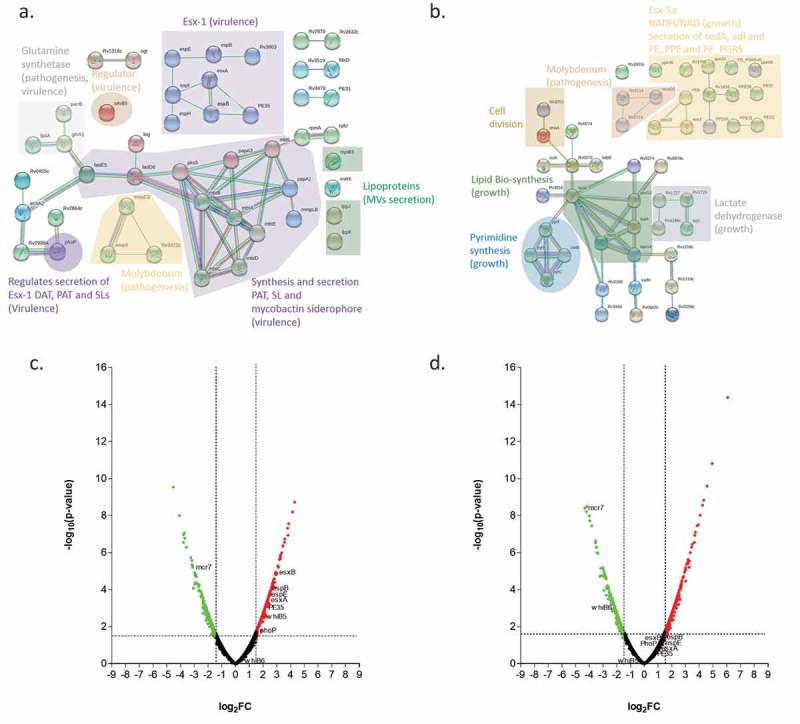
Analysis of differentially expressed genes in Sauton’s medium for UT205 and UT127. (a). STRING analysis of the up-regulated genes for UT205 in Sauton’s medium. (b). STRING analysis of up-regulated genes for UT127 in Sauton’s medium. (c). Volcano plot analysis showing the differentially expressed (DE) genes for UT205 in Sauton’s medium and in (d). showing the DE genes for UT127 in Sauton's medium as well. Logarithm base 2 of the fold change (log_2_FC). Graphs were obtained using the STRING protein-protein interactions networks software version 11.0. The red color (right) is for up-regulated and green (left) for down-regulated genes in Sauton’s medium.

To better understand the functional interactions of the proteins encoded by the transcriptomes of UT127 and UT205 grown under the stressful conditions posed by the Sauton’s medium, we used the STRING database that associates both direct (physical) and indirect (functional) interactions, providing a protein network of genome-wide functional connectivity [36]. For this purpose, we selected the list of upregulated genes expressed by UT127 and UT205. Analyzes of the upregulated genes of the transcriptome of UT205 in Sauton’s medium using the STRING database and focusing on biological processes based on Gene Ontology (GO) categories, showed: (a) the secretion system ESX-1 (*esxA, esxB, espB, espE, espH, espl, PE35* and *rv3863*); (b) the secretion of pathogenic lipid pentacyltrehalose (PAT), sulfolipid-1 (SL-1) (*papA1, papA3 and mmpL8*) (c) mycobactin siderophore (*mbtA, mbtB, mbtC, mbtD, mbtE* and *mbtL*); (d) molybdopterin cofactor (*moaC3, moaX* and *rv3322c*) and finally (e) lipoproteins (*mpt83, lppJ* and *lppK*) (Figure 3(a))(Table 1). The main interaction network in UT205 is comprised by mycobactin genes associated to iron acquisition (*mbtA*-E, *mbtL*) and transport (*mmpL8*) as well as genes associated to lipid biosynthesis, particularly poliketides and fatty acids (*papA1, papA3, pks5, fadD6, fadE5*, and *ech*). Survival and *in vivo* growth of Mtb depends on mycobactins [37]. Interestingly, the *fadE5* gene (*rv0244c*) in addition to *pks5, papA3, pE*, and *fadD23* genes have been recently involved in the synthesis of the complex lipid trehalose polyphleate (TPP) closely related in structure to SL-1 and PAT may be involved in membrane fluidity [38]. The *fadE5* and *pks2* genes were reported to be differentially expressed in the differentiated triglyceride-rich 3T3L1 human adipocyte cell line infected with Mtb H37Rv compared to the non-differentiated cells [39]. Since the *fadE5* gene has been previously found to be essential for *in vitro* growth in cholesterol [40], it has been proposed that upregulation of *fadE5* and *pks2* may be related to the cholesterol-rich environment found in necrotic adipocytes [39]. We also found upregulation of the *fadD6*/FACL6 (*rv1206*) and *echA2* (r*v0456c*) genes in the UT205 strain compared to Mtb H37Rv. It has been previously shown that *fadD6* function as a fatty acid transporter, and its protein level was higher in Mtb H37Rv bacilli in a dormant state than in Mtb in the exponential growth phase [41]. The family of Ech proteins of Mtb is involved in the degradation of cis fatty acids [42]. EchA2 is a monofunctional enoyl CoA isomerase [41] may be involved in unsaturated fatty acid metabolism and essential for Mtb survival in lipid-rich environments [10]. Of note, the PhoP transcription factor upregulation in UT205 may explain upregulation of *pks2, papA1*, and *mmpL8* required for SL synthesis and export of *pks3* and *papA3* involved in DAT/PAT biosynthesis, as well as the upregulation of *esat-6* (*esxA, rv3875*) and *cpf-10* (*esxB, rv3874*) genes [43]. A third gene network of interest is conformed by the *moaC3* (*rv3324c), moaX* (*rv3323c)* and *rv3322c* genes. The genes *moaC3* and *moaX* express proteins associated with the synthesis of the molybdenum cofactor (MoCo) and molybdopterin. MoCo is necessary to catalyze redox reactions in carbon, nitrogen, and sulfur metabolism [44]. MoCo function has been associated with Mtb pathogenesis. Transposon mutagenesis of *moaX* resulted in attenuated growth in macrophages and reduced ability to persist in differentiated THP-1macrophages [45]. Remarkably, the best characterized MoCo-dependent enzyme is NarGHI involved in nitrate respiration and required for survival of Mtb under anaerobic conditions *in vitro* [44]. Clinical isolates of Mtb displayed reduced fitness in macrophages associated with low expression of *narG* in vitro [46].

**Table 1.** Main Gene Ontology (GO) functional categories for UT205 and UT127.

UT205		
GO biological process complete	Observed gene count	FDR
Protein secretion by the type VII secretion system (GO:0044315)	10	2.68E-03
Secretion (GO:0046903)	15	1.18E-02
Peptide secretion (GO:0002790)	15	9.40E-03
Secretion by cell (GO:0032940)	15	7.84E-03
Protein secretion (GO:0009306)	15	6.72E-03
Protein transmembrane transport (GO:0071806)	21	3.41E-02
Pathogenesis (GO:0009405)	166	3.83E-02
UT127		
GO pathway description	Observed gene count	FDR
PPE family	15	0.0106
Penta-peptide repeats (8 copies)	8	0.0106
Proteins of 100 residues with WXG	8	0.0272

In contrast, UT127 showed the following networks: (a) lipid biosynthesis (*fadA, fadB, fabG4, htdX* and *accD2*), (b) lactate dehydrogenase (*lidD, rv1726, rv1727* and r*v1186c*), (c) secretion system ESX-5-ESX-5a (*esxJ, PE8, ppe15, rv1803c, ppe40, ppe24, ppe59, PE22, PE20, PPE31, PPE55, Rv1834* and *PE_PGRS46*) and (d) DNA replication (*dnaA, rv3751, pyrB, pyrC, pyrF* and *carB*) (Figure 3(b)) (Table 1). Two of these networks are composed of genes associated with growth and cell division. The PYR operon encodes the Mtb genes associated with *de novo* pyrimidine biosynthesis, including *pyrB, pyrC*, and *pyrF* as well as *carB* (*rv1384)* involved in the formation of carbamoyl phosphate, the first step in the synthesis of pyrimidines, which is variably expressed by different clinical isolates [47]. The amino acids glutamine and aspartic acid are necessary precursors for the synthesis of the nucleotides. One of the upregulated genes *rv0073* encodes Glutamine-transport ABC transporter ATP-binding protein, a protein importing glutamine, thought to be involved in the active transport of glutamine across the membrane [48]. Two other Mtb genes involved in growth and virulence are upregulated in UT127, *rv3708c* (Aspartate-semialdehyde dehydrogenase) and *rv3709c* (Aspartokinase). The transfer of aspartate-derived nitrogen to glutamate, together with glutamine provides nitrogen to most of the biosynthesis pathways in Mtb [49]. The aspartate metabolism also involves the synthesis of amino acids methionine, threonine and isoleucine involving the ASK and ASD enzymes as well as the synthesis of diaminopimelic acid, an important constituent of the Mtb peptidoglycan [48]. Of note, both glutamine and aspartic acid are involved in the synthesis of pyrimidines [50]. Remarkably, two genes involved in the potassium ion transport system, *kdpD* (*rv1028c*) and *kdpE* (*rv1027c*) [51] are also upregulated in UT127.

Interestingly, the addition of potassium to liquid media containing non-culturable forms of Mtb (not growing in solid medium), resulted in the reactivation of growth of Mtb [52]. The second network is structured with genes participating in the synthesis of fatty acids (*fas, fadA, fadA3, fadB, fadE12, fabG4, accD2*, and *echA19*). Current evidence suggests that fatty acids from host origin are used by Mtb as an energy source for persistence and virulence. Furthermore, the catabolism of host-derived lipids can be used by Mtb to regulate its replication and drug tolerance [53]. In axenic media such as Sauton’s, the primary carbon source for the bacillus is glycerol [54] allowing the growth of Mtb [55]. Thus, in our experimental setting, two of the leading networks observed in UT127 suggest that this strain is responding to the stressful conditions posed by the carbon-poor conditioning by expressing genes associated with replication and growth. We also found a small subnetwork of genes, including *rv1726, rv1727, rv1186*, and *rv1872c*. From these, only the function of Rv1872c (LldD2) is known. LldD2 (possible L-lactate dehydrogenase) has been involved in respiration and catalyzes the conversion of lactate into pyruvate. It has been recently observed that Mtb could use macrophage-derived lactate as a carbon source based on the enzymatic activity of LldD1 (Rv0694). However, the conversion of L-lactate to pyruvate and through gluconeogenesis was strictly dependent on *rv1872c*, suggesting an essential role of lactate oxidation for intracellular growth of Mtb in human macrophages [56]. Notably, Rv1872c has also been involved in holotransferrin iron acquisition [57] and identified as an antigen expressed *in vivo* in lungs from infected mice [58].

Finally, the largest upregulated gene network groups genes of the PE and PPE repetitive gene families suspected to be involved in different aspects of the immune response, including cell death and T-cell responses [59]. However, the specific function of most of them is unknown. We found upregulation of genes in UT127 related to PE, PPE, and PE_PGRS proteins, which have been showed to be secreted through the ESX-5 secretion system. It is known that the ESX-5 system is required for the activation of the host cell inﬂammasome and consequently, IL-1β secretion by Mtb infected cells [60]. Furthermore, it has been showed that the ESX-5 secretion system induces a caspase-independent form of cell death in macrophages infected with Mtb [60]. We also found genes related to a paralog region of ESX-5 called ESX-5a that allows the secretion of some PE/PPE proteins as well as two important proteins: alanine L-dehydrogenase (ALD) and superoxide dismutase A (SodA) [61].

An important result is the significantly higher upregulation of the main components of the ESX-1 secretion system and the regulatory protein PhoP that was found in UT205 as compared to UT127, which is shown in the volcano plots (Figure 3(c,d)). The ESX-1 system is one of the main differentiation factors that may be determining a big percentage of the more virulent phenotype observed in the clinical isolate UT205. It has been reported that the ESX-1 secretion system can be induced by growing the mycobacteria in Sauton’s medium [62–64]. The ESX-1 secretion system is a region that is absent in BCG, and it has been involved in virulence through proteins such as Esx-A (ESAT6) and Esx-B (CFP-10) [65]. The protein Esx-A has multiple effects during Mtb infection, one of them is the induction of membrane lysis that has been associated with the escape of Mtb from the phagosome into the cytoplasm that may cause macrophage cell death [12]. Moreover, Esx-A can inhibit antigen-presentation by the inhibition of TLR signaling pathways that reduce the IL-12 production [66]. The ESX-1 system is necessary for the secretion of Esp (ESX-1 secretion-associated protein) proteins such as *EspA-C, EspE*, and *EspJ* [67,68].

In addition to the previous comparisons between UT127 and UT205 with H37Rv, we did a direct comparison between UT205-7H9 and UT205-Sauton’s medium in order to gather information of additional pathways, involved in the observed growth changes of UT205 in the two different types of medium (Supplementary Figure 4(a,b)). We used STRING for this comparison using the up-regulated and down-regulated DE genes (log_2_FC>2 and p-value>0.05) (Supplementary Table 6). The data showed that UT205-Sauton’s increase in the metabolism of lipids and cholesterol. Moreover, the data support that UT205 may produce more mycolic acids, PAT and DAT, as well as other virulence factors such as the ESX-1 system in the Sauton´s medium as compared to the 7H9 (Supplementary Figure 4(a)). Interestingly, there is a downregulation in the production of PGL when UT205 was grown in Sauton’s medium as compared to 7H9 medium (Supplementary Figure 4(b)). These observations confirm the previous findings using the comparisons with the control strain H37Rv. For additional support of the data showed in the paper, we validate some of the most up-regulated and downregulated genes of the RNAseq data by qRT-PCR (Supplementary Figure 4(c)).

### sRNAs changes in the transcriptomes

Small noncoding regulatory RNAs (sRNAs) have proven to be effective, and central regulators that play roles in stress responses, controlling virulence genes as well as oxidative and hypoxic stresses responses [69]. We found 31 differentially expressed sRNAs in the transcriptomes of UT127 and UT205 under the growth conditions of 7H9 and Sauton’s substrates (Table 2). From these 31 sRNAs, 21 corresponded to UT127 7H9, 9 sRNAs for UT205 in 7H9, 15 sRNAs for UT127 in Sauton’s and 7 sRNAs for UT205 in Sauton’s. Particularly, the sRNAs *mpr6* and *mcr7* were downregulated in all conditions for both clinical isolates. The sRNA *B11* was upregulated in all conditions except for UT205-Sauton’s, and the sRNA *ncrMT1234* was downregulated in all conditions except for UT205-Sauton’s media. The sRNA *MTS2823* was downregulated only in UT127 in 7H9 and then upregulated when it was grown in Sauton’s. The sRNAs *ncRv3583A, mpr17, ncRv11435c, mcr16, ncRv10537A, ncRv11248c, F6, ncRv11414Ac, ncRv0412A, ncRv0932A* and *MT3949* were upregulated exclusively in UT127-7H9 and the sRNA *ncRv13003Ac* was exclusively downregulated under the same condition. Also, the sRNAs *mpr12, ncRV12659, ncRv11174Ac* and *ncRv13241Ac* were downregulated only in UT127-Sauton’s. In a direct comparison between UT127-7H9 and UT205-7H9, we found that *mcr11* was 3 times more expressed in UT205.

**Table 2.** Differentially expressed sRNAs for UT127 and UT205.

	UT127 7H9		UT205 7H9		UT127 Sauton's		UT205 Sauton's	
sRNA gene	log_2_FC	p-value	log_2_FC	p-value	log_2_FC	p-value	log_2_FC	p-value
*ncRv3583A*	**7,08**	2,90E-18						
*mpr17*	**6,08**	3,82E-14						
*ncRv11435c*	**5,96**	9,93E-14						
*mcr16*	**5,52**	8,04E-13						
*ncRv10537A*	**4,13**	8,47E-09						
*G2*	3,68	1,98E-07					1,59	1,90E-02
*ncRv11248c*	**3,44**	8,73E-07						
*F6*	**2,87**	1,83E-05						
*ncRv11147Ac*	2,61	8,05E-05	2,03	1,73E-03				
*ncRv11414Ac*	**2,31**	7,41E-04						
*MTS1338*	2,13	1,26E-03			3,34	1,00E-06	1,85	4,40E-03
*ncRv0412A*	**2,09**	1,47E-03						
*ncRv0932A*	**2,01**	1,75E-03						
*ncrMT3949*	**1,8**	4,86E-03						
*B11*	**1,5**	1,85E-02	**1,71**	7,83E-03	**1,86**	3,86E-03		
*ncRv13003Ac*	−1,81	5,11E-03						
*MTS2823*	**−1,85**	3,73E-03			**2,74**	3,38E-05		
*mpr6*	**−2,32**	4,89E-04	**−1,66**	1,07E-02	**−3,38**	1,08E-06	**−3,17**	4,46E-06
*mcr3*	−2,54	1,05E-04			−2,85	1,79E-05		
*ncrMT1234*	**−3,07**	5,87E-06	**−3,54**	3,16E-07	**−2,77**	2,01E-04		
*mcr7*	**−4,07**	2,15E-08	**−2,4**	3,02E-04	**−4,33**	3,89E-09	**−3,12**	6,25E-06
*MTS0858*			2,19	1,43E-03	2,21	1,65E-03	2,07	3,17E-03
*ncRv2345Ac*			−2,04	2,11E-03				
*ncRv13385Ac*			−1,54	2,35E-02	−1,63	2,53E-02		
*ncRv3418Ac*			1,66	9,14E-03				
*mpr12*					**−2,94**	1,09E-04		
*MTS1082*					2,99	8,42E-06	1,74	6,84E-03
*ncRv12659*					**2,47**	1,62E-04		
*ncRv11075A*					2,16	1,03E-03	1,88	4,02E-03
*ncRv11174Ac*					**1,72**	7,73E-03		
*ncRv13241Ac*					**1,69**	8,06E-03		

The function and mechanism of action of the Mtb sRNAs are mostly unknown and especially for the majority of the ones that were induced by both clinical isolates. Called our attention, the sRNA *mcr16* was induced only in UT127-7H9 (log_2_FC 5.5). It has been suggested that *mcr16* could regulate the *fabD* (*rv2243*) gene that functions as a Malonyl CoA-acyl carrier protein transacylase that play an essential role in the FASII lipid synthesis system and is essential in Mtb [70]. Interestingly, our data show that the expression of *fabD* is upregulated in UT127-Sauton’s but is not in UT127-7H9. Also, *fabD* appears as a central protein in the upregulated network for UT127-Sauton’s. It is known that sRNAs can modify the gene expression either promoting or inhibiting the production of bacterial proteins that include various effector molecules as well as proteins necessary for the adaptive responses of bacteria against environmental changes.

## Discussion

*In vitro* culture of Mtb under conditions restricting survival and/or proliferation such as hypoxia, acidic pH, and nutrient deprivation, among others, which happen during macrophage infection, has been used to find critical genes associated to virulence and pathogenicity [71,72]. A carbon-restricted medium such as Sauton’s has been used to simulate particular stress conditions found by Mtb during its residency in phagocytic cells or inside the granuloma. Some evidence indicates that the culture of mycobacteria in Sauton’s medium leads to a particular adaptive response compared to a less carbon-restricted medium such as 7H9 [62,73–75]. Also, the growth of *M. bovis* BCG in Sauton’s medium conferred resistance to BCG against killing by primary macrophages, which correlated with an ability to grow and persist longer in mice when compared to the same BCG strain grown in Middlebrook 7H9 medium [76].

The depiction of virulence determinants of Mtb circulating strains relevant to TB human disease is critical to achieving a better understanding of the pathogenesis of TB. During recent years, the TB field is reaching an agreement to affirm that this disease is a complex spectrum of infection outcomes in which the circulating strains are one of the crucial components [5]. This heterogeneity of the Mtb circulating strains occurred at the population level but also in the lungs of a single infected individual [77], which is reflected in the diversity, progression, and resolution of its lung granulomas. At the TB affected populations, it has been shown that the differences in the severity of the disease, transmission potential, and curation rates are mostly associated with virulence factors and mutations that cause antibiotic resistance. Some of the virulence factors associated with differences in immunopathology have been associated with differences in the production of distinct cell wall glycolipids, lipids with long chain fatty acids together with specific proteins and their secretion systems [5,65].

The main phenotypic differences that we found, among the two Colombian clinical isolates of Mtb UT127 and UT205, are related to growth kinetic and MVs secretion when they are grown in Sauton´s medium, which is to our knowledge the first report of a significant difference in the MVs production among clinical isolates. Notably, the transcriptome of UT205 grown in Sauton’s medium showed that the predominant pathways for this clinical isolate are mainly associated with virulence; in contrast, UT127 showed pathways mainly associated with growth and survival. The results of our transcriptional studies, sustain the idea that these two Mtb closely related circulating strains differ in their virulence programs. Collecting evidence from different Mtb clinical isolates, as well as our results, suggests that transcriptomic, genetic differences may account for relevant phenotypic manifestations that could affect the outcome of infection [78]. A recent paper in which a panel of clinical isolates was compared for gene essentiality, establish that certain genes were differentially required in only a subset of strains but not in others, which could be an adaptation of the strains to do specific metabolic processes [79]. Another study in which clinical isolates from Brazil were compared in their growth kinetics, determined very heterogeneous rates of growth, which could reflect differential programs of adaptation to the culture conditions [80].

The result of the transcriptomes reported in this paper, in which is evident the reduction in the amount of upregulated genes of UT127 in 7H9 as compared to Sauton’s media, may account for a most stressful condition for UT127 in contrast to UT205 in which the number of upregulated genes did not change dramatically between the two conditions. In an exciting publication [81], in which chromatin immunoprecipitation (ChIP-on-chip) and microarray analysis were used, it was determined that the alternative sigma factor SigF (Rv3286) directly regulate genes encoding proteins involved in lipid synthesis, intermediary metabolism, and virulence. It has been shown that SigF is expressed during nutrient starvation [82]. Moreover, the deficiency of SigF leads to partial attenuation of Mtb in animal models [83,84]. Interestingly, we found that *sigF* was three times more expressed in UT127-7H9 than UT127-Sauton’s, a result that is counterintuitive because it is the opposite of what we would expect. This reduction in the expression of sigF may be associated with the decrease in the fitness of UT127 when it is growing under a nutrient deprivation condition.

In order to get a good profile from the data, we frame the more virulent transcriptomic profile of UT205 into two main aspects that confer a better fitness to this clinical isolate. First, the relationship between MVs secretion, mycobactins and iron acquisition, and second, the expression of the ESX-1 system, the lipid secretion and its regulation by PhoP. Iron is a well-recognized and essential element, required for Mtb viability and survival during the host infection, as well as for pathogenesis. The capability of mycobacteria to compete for iron, may influence the progress of the disease and host-pathogen interactions. It was recently demonstrated that the rate of MVs production increases in an iron-limited medium such as Sauton´s medium [85].

Moreover, it was also showed that MVs contain mycobactins which may escalate the effectiveness of siderophore production during iron limitation. MVs have been implicated in immunomodulation through the export of agonist that activates the TLR-2 pathway [16,30]. Also, it has been suggested that MVs may be part of an immune evasion strategy of Mtb that supports its dissemination in the host. The second framework, which relates to the higher up-regulation of the main components of the ESX-1 secretion system in UT205 as compared to UT127, is in resonance with a report that showed that different clinical isolates isolated from patients, express differential levels of the ESX-1 secretion system [86]. In line with the previous, it is known for some time that Mtb uses the ESX-1 system to disturb the phagosomal membrane in order to escape to the cytoplasm which allows the bacteria to disseminate. The mycobacterial ESX-1 secretion system has been associated with exacerbated virulence and pathogenicity. Moreover, PhoP is a master regulator of different genetic networks controlling the ESX-1 secretion system [43]. PhoP binds to the promoter region of the gen *espR*, which has been proposed as a nucleoid-associated protein that regulates the EspACD operon. Additionally, PhoP regulates the expression of the transcription factor WhiB6, by interacting with its promoter region, and then this factor regulates the expression of more components of the secretion system. PhoP-controlled genes are responsible for the production of SL and DAT/PAT, such as *pks2, papA1*, and *mmpL8*. It has been suggested that DAT/PAT, SL-1, and PDIM collectively contribute to a reduction of the immune response to Mtb. Disruption of the PhoPR system markedly affects the ability of Mtb to replicate in cellular and animal models.

**Figure 4.**
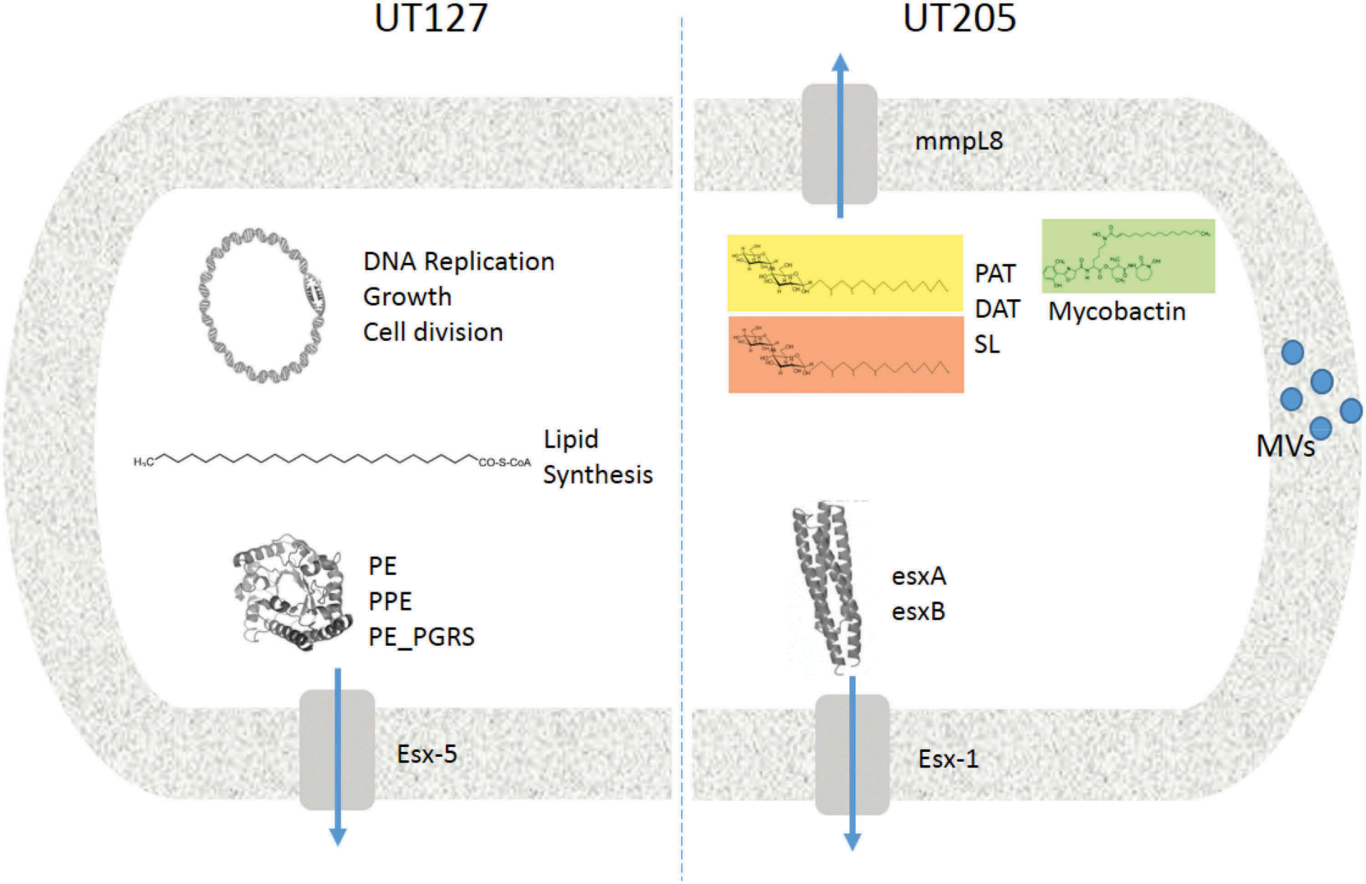
Comparative model of the main virulence factors associated with UT127 and UT205 in Sauton’s medium. Representative diagram to show the differences between UT127 and UT205. The main pathways that are activated by UT127 are more related to survival while for UT205 are more related to virulence.

In conclusion, the transcriptome analysis presented in this paper showed that UT127 and UT205 were significantly different when they were grown under different carbon and energy sources. A close inspection of the network interactions of differentially expressed upregulated genes in UT127 and UT205 Mtb strains cultured in Sauton’s media evidence two different genetic programs in response to the restriction imposed by the medium (Figure 4). Based on our previously published findings in which some Mtb strains of the LAM family may have more transmissibility in Colombia [13] and that this may be associated with increased virulence of these clinical isolates [12], our data contribute to the understanding of the heterogeneity in the circulating strains that cause TB in Colombia. We did not find critical genetic differences between the two clinical isolates UT127 and UT205, and we cannot associate the phenotypic differences to a single genomic feature accounting for potential differences in virulence and transmission of these Mtb clinical isolates. Additionally, we cannot rule out the possibility that epistatic interactions between many different genes or epigenetic changes in promoters or other regulatory regions that affect gene expression at specific loci, which could be responsible for the phenotypic differences observed between these clinical isolates.

Given the success of Mtb as a pathogen, it is not surprising that Mtb can use different genetic regulatory programs to control its growth as a survival strategy. Furthermore, the findings of this paper may contribute with information about the diversity of genetic programs that are present in the multiple Mtb strains that are currently circulating in the TB affected populations around the world. Future considerations of the potential consequences of strain variation should be taken more seriously, in order to search for better antibiotics and vaccines against TB.
